# 1380. Machine Learning-based Estimation of Unconfirmed COVID-19 Cases from a 10,000-Household Survey in Gilgit-Baltistan, Pakistan

**DOI:** 10.1093/ofid/ofad500.1217

**Published:** 2023-11-27

**Authors:** Daniel S Farrar, Lisa G Pell, Yasin Muhammad, Sher Hafiz, Lauren Erdman, Diego G Bassani, Zachary Tanner, Imran Ahmed, Karim Muhammad, Falak Madhani, Shariq Paracha, Masood Ali Khan, Sajid B Soofi, Monica Taljaard, Rachel Spitzer, Sarah M Abu Fadaleh, Zulfiqar A Bhutta, Shaun Morris

**Affiliations:** Centre for Global Child Health, The Hospital for Sick Children, Toronto, Ontario, Canada; The Hospital for Sick Children, Toronto, Ontario, Canada; Aga Khan Health Service, Pakistan, Gilgit, Northern Areas, Pakistan; Aga Khan Health Service, Pakistan, Gilgit, Northern Areas, Pakistan; The Hospital for Sick Children and University of Toronto, Toronto, Ontario, Canada; The Hospital for Sick Children, Toronto, Ontario, Canada; The Hospital for Sick Children, Toronto, Ontario, Canada; Aga Khan University, Karachi, Sindh, Pakistan; Aga Khan University, Karachi, Sindh, Pakistan; Aga Khan Health Service, Pakistan, Gilgit, Northern Areas, Pakistan; Aga Khan Health Service, Pakistan, Gilgit, Northern Areas, Pakistan; Aga Khan Health Service, Pakistan, Gilgit, Northern Areas, Pakistan; Aga Khan University, Karachi, Sindh, Pakistan; Ottawa Hospital Research Institute, Ottawa, Ontario, Canada; The Hospital for Sick Children, Toronto, Ontario, Canada; The Hospital for Sick Children, Toronto, Ontario, Canada; The Hospital for Sick Children, Toronto, Ontario, Canada; Hospital for Sick Children, University of Toronto, Toronto, Ontario, Canada

## Abstract

**Background:**

Robust estimates of COVID-19 prevalence during the pandemic are scarce, particularly in settings with limited SARS-CoV-2 testing. Gilgit-Baltistan (GB) is a remote region of Pakistan where healthcare access is limited by underdeveloped facility and road infrastructure. We leveraged a large household survey to describe the burden of confirmed and unconfirmed COVID-19 in GB.

**Methods:**

We conducted a cross-sectional survey in GB from June–August 2021 during the baseline phase of a cluster randomized trial. Households were randomly selected using a stratified, two-stage sampling design. Data regarding SARS-CoV-2 testing, healthcare worker (HCW) diagnoses without testing, symptoms, and outcomes since March 2020 were self-reported for all household members. “Confirmed/probable” COVID-19 was defined as a positive test, HCW diagnosis of COVID-19, or HCW diagnosis of pneumonia with COVID-19 positive contact. Using machine learning (ML) and bootstrap validation, we developed a symptom-based diagnostic model to differentiate confirmed/probable infections from those with negative SARS-CoV-2 tests (Fig. 1). We applied this model to untested respondents to estimate the total prevalence of COVID-19.

**Figure 1.**
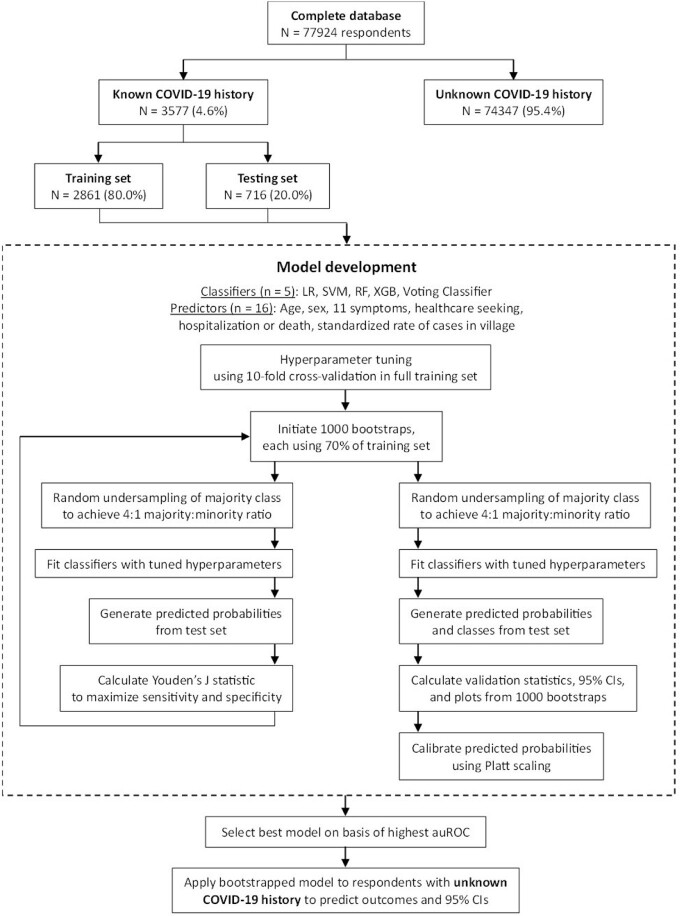
Workflow diagram for machine learning analysis. auROC=Area under the receiver operating characteristic curve; CI=Confidence interval; LR=Logistic regression; RF=Random forest; SVM=Support vector machines; XGB=eXtreme Gradient Boosting

**Results:**

Data were collected from 77924 people in 10264 households. Overall, 314 had confirmed/probable COVID-19, 3263 had negative tests, and 74347 were untested. SARS-CoV-2 testing was less common in females (vs. males; 38 vs. 58 tests per 1000 people) and children (vs. adults; 17 vs. 76 tests per 1000 people). Using an extreme gradient boosting model, area under the receiver operating characteristic curve was 0.92 (95% confidence interval [CI] 0.90–0.93), sensitivity was 0.81 (CI 0.75–0.85), and specificity was 0.88 (CI 0.85–0.90). With this model, total estimated cases were 8–17 times more than the number of individuals with positive tests (Fig. 2). The ratio of estimated to confirmed cases was higher for children (90–213 times) and females (13–25 times).

**Figure 2.**
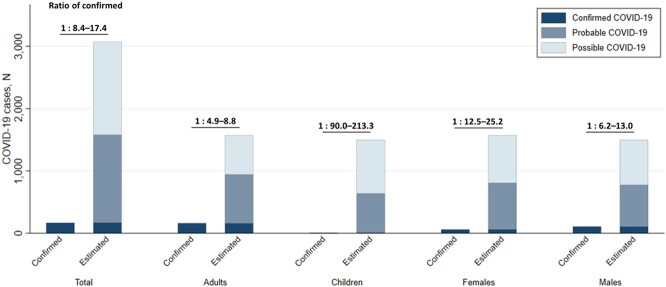
Estimation of probable and possible COVID-19 cases, overall and by age and sex. Confirmed COVID-19 indicates individuals with positive SARS-CoV-2 tests; probable COVID-19 includes HCW diagnoses of COVID-19 and positive predictions from the machine learning analysis; possible COVID-19 includes individuals with positive close contacts or HCW diagnoses of pneumonia. Ratios are depicted as a plausible range between ‘confirmed : probable’ and ‘confirmed : probable + possible’.

**Conclusion:**

From March 2020–August 2021, the majority of COVID-19 cases in GB went unconfirmed. Women and children were tested less often, perhaps due to preferences in healthcare seeking and perceptions of lower risk of severe illness. Our approach may be used to estimate COVID-19 prevalence in settings with limited testing capacity.

**Disclosures:**

**Shaun Morris, MD, MPH, DTM&H, FRCPC, FAAP**, GlaxoSmithKline: Honoraria|JNJ China: Honoraria|Merck: served on ad hoc advisory board|Pfizer: Grant/Research Support|Pfizer: served on ad-hoc advisory board|Sanofi-Pasteur: served on ad-hoc advisory board

